# The point-of-care D-dimer test provides a fast and accurate differential diagnosis of Stanford Type A aortic syndrome and ST-elevated myocardial infarction in emergencies

**DOI:** 10.1186/s12872-022-02925-x

**Published:** 2022-12-21

**Authors:** Xiaoxin Chang, Jianhua Yao, Yawei Xu

**Affiliations:** Department of Cardiology, Tongji University, Shanghai Tenth People’s Hospital, Tongji University School of Medicine, 301 Yanchang Road, Shanghai, 200072 China

**Keywords:** Stanford type A aortic syndrome, Coronary reperfusion therapy, D-dimer, ST-segment elevated myocardial infarction

## Abstract

**Background:**

The research of the sensitivity and specificity point-of-care testing (POCT) of D-dimer as a diagnostic protocol for differential diagnosis of Stanford Type A aortic syndrome (hereafter as TAAS) mimicking ST-elevated myocardial infarction (STEMI) with regular STEMI in the emergency department is limited.

**Methods:**

Full medical information of 32 patients confirmed TAAS and 527 patients confirmed STEMI from January 1st, 2016 to October 1st, 2021 were retrospectively analyzed in Shanghai Tenth People’s Hospital of Tongji University.

**Results:**

The baseline characteristics of two groups of patients were well-balanced post propensity score matching (PSM) analysis, and each group had 32 patients enrolled. Patients in the STEMI group had higher positive cardiac troponin I (cTNI) (0.174 ng/ml vs. 0.055 ng/ml, *P* = 0.008) results but lower D-dimer (0.365μg/ml vs. 31.50μg/ml, *P* < 0.001) results than the TAAS group. The D-dimer cutoff value of 2.155μg/ml had the best sensitivity of 100% and specificity of 96.9%, and the positive predictive value (PPV) as well as the negative predictive value (NPV) of the cutoff value were 96.9 and 100%, respectively, in total 64 patients, the area under the curve (AUC) values were 0.998 (95% CI:0.992-1.000, *P* < 0.001) for the D-dimer. No significant correlation between the D-dimer concentration and the time from symptoms onset to first medical contact in both groups (TAAS group: r = − 0.248, *P* = 0.170; STEMI group: *r* = − 0.159, *P* = 0.383) or significant correlation between D-dimer and creatine clearance (TAAS group: *r* = − 0.065, *P* = 0.765; STEMI group: *r* = 0.222, *P* = 0.221). The total in-hospital mortality for the patients with TAAS presenting as STEMI was 62.5% (20/32). The mortality rate for TAAS patients confirmed by computed tomography angiography (CTA) was significantly lower (40% vs. 82.4%, *P* = 0.014) than the mortality rate for TAAS patients confirmed by coronary angiography (CAG) and had a longer average survival time (log-rank = 0.015), less peri-surgical complications especially gastrointestinal hemorrhage (0.00% vs. 55.6%, *P* < 0.001). CTA diagnosis can reduce the mortality rate by 67.5% (95%CI:0.124-0.850, *P* = 0.16).

**Conclusions:**

The POCT D-dimer with cut-off 2.155μg/ml would be useful to rule-out TAAS mimicking STEMI from regular STEMI prior to reperfusion therapy. CTA diagnosis is effective in reducing the probability of perioperative complications and lowering perioperative mortality than CAG diagnosis in TAAS patients.

**Supplementary Information:**

The online version contains supplementary material available at 10.1186/s12872-022-02925-x.

## Introduction

Stanford type A aortic syndrome (TAAS) extends to the ostium of the coronary artery may lead to acute coronary occlusion, thus leading to acute coronary syndrome (ACS) and mimicking as STEMI in electrocardiography examination. It is a life-threatening acute vascular disease that is not easily detectable because the primary electrocardiographic (ECG) presentation will make physicians ignore the atypical clinical manifestations and lead to misdiagnosis in the Emergency department (ED) [1]. Methods include computed tomography (CT), transesophageal echocardiography (TEE), transthoracic echocardiography (TTE), magnetic resonance imaging (MRI), and point-of-care ultrasound (POCUS) are used to exclude the TAAS in STEMI patients in clinical practice when there is a suspicion [2]. While in ED, TAAS might be ignored and misdiagnosed because definitive imaging may delay the reperfusion time and regular STEMI happens more often. Therefore, it is crucial to find a reliable, fast method to differentiate whether the STEMI is secondary to TAAS and to reduce misdiagnosis. Previous studies have found that D-dimer concentrations can be elevated in TAAS [3], despite its low specificity for the condition, we found that POCT of D-dimer is a simple and efficient method for detecting and discriminating acute aortic syndrome with ST-elevated myocardial infarction in the ED and thus leading to the important clinical decision making.

Here, we retrospectively analyzed the clinical characteristics, lab tests, and outcomes of the confirmed patients diagnosed with STEMI and STEMI secondary to TAAS, hoping this study could provide deep insight into such conditions and offered an effective and fast method to reduce such misdiagnosis and improve the prognosis of these patients.

## Materials and methods

### Patients

This is a one-center retrospective study. 34 patients who confirmed STEMI secondary to TAAS and 551 STEMI patients in ED enrolled in the study between January 1st, 2017 and October 1st 2021 were evaluated. 24 STEMI patients and 2 TAAS patients were excluded from the study for the lack of the POCT of D-dimer or cardiac infarct biomarker results in the ED.

Stanford type A aortic syndrome was defined as intramural hematoma or aortic dissection involvement of the ascending aorta and aortic arch. Enhanced computed tomography (CTA) or coronary angiography (CAG) confirmed the diagnosis of TAAS.

The diagnosis of STEMI was based on the latest criteria established by the American College of Cardiology [4] and European Society of Cardiology [5], including: (1) chest pain lasting for over 30 min, (2) at least two contiguous leads with ST-segment elevation 2.5 mm in men < 40 years, 2 mm in men over 40 years, or 1.5 mm in women in leads V2–V3 and/or1mm in the other leads, (3) an increase of cardiac biomarker values with at least one value above the 99th percentile of the upper reference limit. And other ECG signs of coronary artery occlusion like De winter syndrome were also included.

### Data collection

After approval by the Clinical Ethics Committee (CEC) of the Shanghai Tenth People’s Hospital (application number: SHYS-IEC-5.0/22 K220/P01), two independent physicians retrospectively collected and reviewed the general clinical information, demographics, treatment records, and essential time points according to standards. POCT results, including cTNI, myoglobin (Myo), creatine kinase–myocardial band (CK-MB), brain natriuretic peptide (BNP), D-dimer (DD) were recorded.

The D-dimer assay was performed with the TRIAGE platform assay panels (Alere, San Diego, CA, USA), which comprises a fluorescence immunoassay analyzer and reagents. The D-dimer results were available within 15 minutes after the platform assay panels being placed on the cardiac reader. The following assay results were predefined to be positive on either blood draw: troponin I 0.05 ng/ml or greater, CK-MB 4.3 ng/ml or greater, Myo concentration of 108 ng/ml or greater, BNP 100 pg/ml or greater, and D-dimer 0.6μg/ml or greater. The point cutoffs were based on manufacturer recommendations, with an elevated result defined as any detectable concentration of results.

### Statistical analysis

The frequency and percentage of the categorical data were determined. Continuous variables were presented as the 25 and 75% percentiles, median, mean and standard deviation (SD), depending on whether the data were normally distributed. A Mann-Whitney U test, chi-square, the Fischer exact or t-test were used to analyze the difference. A value of *p* < 0.05 was considered being significant. Propensity score matching (PSM) was used for calculating variables included sex, age, hypertension, coronary heart disease, smoking, diabetes mellitus, Marfan syndrome, previous percutaneous coronary intervention (PCI), previous coronary artery disease, previous myocardial infarction, known aneurysm, and hyperlipidemia to balance heterogeneity in demographics. The 1:1 PSM was applied to create the matched TAAS group and STEMI group with a caliper distance of 0.1. After PSM, the differences between the two groups were compared again using the aforementioned statistical methods.

Receiver operating characteristic (ROC) curves were constructed after calculation of the sensitivity for the matched TAAS group to determine the best cutoff value with a 95% confidence interval (CI) and the sensitivity, specificity, positive predictive value (PPV), and negative predictive value (NPV) were calculated based on several cut-off values of D-dimer. The Kaplan-Meier estimates of the mortality rates in both groups are reported along with the corresponding hazard ratios (HR) and 95% CIs. All statistical analyses were performed using SPSS Statistics, version 26 (Armonk, NY, USA).

## Results

### Baseline Characteristics

Detailed demographics are displayed in Table 1. 527 STEMI patients and 32 TAAS patients were finally enrolled in the study from January 1st, 2016 to October 1st, 2021. Male patients accounted mainly in both TAAS and STEMI groups (78.1% vs. 82.0%, *P* = 0.584). Compared with the STEMI group, TAAS group had similar age (64[59-66] years vs. 66[61-73] years, *P* = 0.130), similar proportions of smokers (90.6% vs. 90.5%, *P* = 0.983), of patients with previous history of PCI (12.5% vs. 13.3%, *P* = 0.899), of patients with hyperlipidemia (56.3% vs. 53.7%, *P* = 0.779), of patients with hypertension (87.5% vs. 90.7%, *P* = 0.548), and of patients with coronary heart disease (25.0% vs. 26.6%, *P* = 0.845), but of more patients with known aortic aneurysm (9.4% vs. 2.8%, *P* < 0.05) based on the medical history. After 1:1 PSM analysis, each of 32 patients in the TAAS-group and the STEMI-group was extracted, and baseline characteristics were well balanced between the two groups.

**Table 1:** Demographics and characteristics of the patients

Variable	Pre-PSM			Post-PSM		
	TAAS group (*n =* 32)	STEMI group (*n =* 527)	P- value	TAAS group (*n =* 32)	STEMI group (*n =* 32)	*P*-value
Age (years)	64 [59,66]	66 [61,73]	0.130	64 [59,66]	69 [59.5,75.5]	0.053
Men	25 (78.1%)	432 (82.0%)	0.584	26 (81.3%)	25 (78.1%)	0.756
History of tobacco (n, %)	29 (90.6%)	477 (90.5%)	0.983	29 (90.6%)	29 (90.6%)	1
Diabetes mellitus	7 (21.9%)	154 (29.2%)	0.719	13 (40.6%)	9 (28.1%)	0.292
Referred from subordinate hospitals	6 (18.8%)	108 (20.5%)	0.812	6 (18.8%)	6 (18.8%)	1
First consultation at our hospital	26 (81.3%)	445 (79.5%)	0.812	26 (81.3%)	26 (81.3%)	1
Risk factors and characteristics (n, %)						
History of hypertension	28 (87.5%)	478 (90.7%)	0.548	28 (87.5%)	28 (87.5%)	1
Marfan syndrome	0 (0%)	0 (0%)	1.000	0 (0%)	0 (0%)	1
Known aortic aneurysm	3 (9.4%)	15 (2.8%)	0.042	1 (3.1%)	3 (9.4%)	0.302
Previous history of PCI	4 (12.5%)	70 (13.3%)	0.899	4 (12.5%)	4 (12.5)	1
Previous atherosclerostic coronary artery disease	8 (25.0%)	140 (26.6%)	0.845	9 (28.1%)	8 (25%)	0.777
Peripheral artery disease	1 (3.1%)	25 (4.7%)	1.000	1 (3.1%)	1 (3.1%)	1
Previous myocardio infarction	0 (0.0%)	12 (2.3%)	0.814	0 (0%)	0 (0%)	1
Renal dysfunction	1 (3.1%)	32 (5.9%)	0.492	2 (6.3%)	1 (3.1%)	0.554
Hyperlipidemia	18 (56.3%)	283 (53.7%)	0.779	18 (56.3%)	18 (56.3%)	1
Cold sweats	26 (81.3%)	406 (77%)	0.581	26 (81.3%)	26 (81.3%)	1

Data are presented as the median [interquartile range] or no. (%) of patients

### Clinical Data

The clinical characteristics of these patients were also summarized in Table 2. Anterior chest pain was the most common initial presenting symptom among the TAAS and STEMI groups (28.1% vs. 30.2%, *P* = 0.226), subsequent by syncope (25.0% vs. 19.4%, *P* = 0.435) in PSM analysis. In the TAAS group, 7 (21.9%) patients also suffered from abdominal as well as anterior chest pain, 4 (12.5%) were sent to the hospital for cardiogenic shock and 1 (3.1%) had no other symptoms except abdominal pain. Differences in initial symptoms’ presentations were not significant between the 2 groups, and patients in both groups had normal blood pressure (105 mmHg vs. 105 mmHg, *P* = 0.543). Even patients in the STEMI group had more heartbeats (78 bpm vs. 60 bpm, *P* = 0.011) per minute, the result was of no clinical specificity.

**Table 2:** Clinical symptoms and ECG presentations

Variable	Pre-PSM			Post-PSM		
	TAAS group (*n =* 32)	STEMI group (*n =* 527)	P- value	TAAS group (*n =* 32)	STEMI group (*n =* 32)	*P*-value
Clinical symptoms						
Abdinal pain + syncope	0 (0%)	11 (2.1%)	1	0 (0%)	1 (3.1%)	1
Anterior chest pain	9 (28.1%)	159 (30.2%)	0.806	9 (28.1%)	5 (15.6%)	0.226
Posterior chest pain	0 (0%)	11 (2.1%)	0.409	0 (0%)	1 (3.1%)	1
Back pain + syncope	0 (0%)	5 (0.9%)	1	0 (0%)	0 (0%)	1
Abdomina pain	1 (3.1%)	29 (5.5%)	0.865	1 (3.1%)	3 (9.4%)	0.606
Chest pain + back pain	1 (3.1%)	9 (1.7%)	1.0	1 (3.1%)	0 (0%)	1
Cardiogenic shock	4 (12.5%)	45 (8.5%)	0.442	4 (12.5%)	3 (9.4%)	1
Chest pain + syncope	2 (6.3%)	24 (4.6%)	0.992	2 (6.3%)	3 (9.4%)	1
Syncope	8 (25.0%)	102 (19.4%)	0.435	8 (25%)	9 (28.1%)	0.777
Persistent or intermittent chest tightness	0 (0%)	30 (5.7%)	0.325	0 (0%)	4 (12.5%)	0.113
Anterior pain + abdmina pain	7 (21.9%)	98 (18.6%)	0.645	7 (21.9%)	3 (9.4%)	0.302
Different presentations of ST-segments elevation in ECG						
II, III, AVF leads	20 (62.5%)	201 (38.1%)	0.006	20 (62.5%)	13 (40.6%)	0.08
V1-V3 leads	2 (6.3%)	45 (8.5%)	0.651	2 (6.3%)	3 (9.4%)	1.000
V1-V5 leads	5 (12.5%)	31 (5.9%)	0.261	4 (12.5%)	1 (3.1%)	0.352
V3-V5 leads	0 (0%)	190 (36.1%)	0.000	0 (0%)	13 (40.6%)	0.000
V7-V9 leads	0 (0%)	4 (0.8%)	1.000	0 (0%)	2 (6.3%)	0.492
V3R-V4R leads	0 (0%)	7 (1.3%)	1.000	0(%)	0 (0%)	1
AVR lead	1 (3.1%)	16 (3.1%)	1.000	1 (3.1%)	0 (0%)	1
I, AVL leads	0 (0%)	25 (4.7%)	0.388	0 (0%)	0 (0%)	1
De Winter Syndrome	1 (3.1%)	7 (1.3%)	0.949	1 (3.1%)	0 (0%)	1
New onset LBBB	0 (0%)	1 (0.2%)	1.000	0 (0%)	0 (0%)	1
Physical examination						
Systolic pressure	105 [100,124]	105 [100,120]	0.415	105 [100,124]	105 [95,122]	0.543
Diastolic pressure	60 [55,65]	60 [55,65]	0.676	60 [55,65]	57 [54,65]	0.566
Heart rate	60 [51.5,85]	78 [75,84]	0.000	60 [51.5,85]	78 [71,83]	0.011
Respiration rate	22 [20,23.5]	22 [18,23]	0.042	22 [20,23.5]	22 [18,23]	0.237

Data are presented as the median [interquartile range] or no. (%) of patients

The median time of symptoms onset to first medical contact was 85 minutes and 180 minutes and the median first medical contact time to ECG examination time was 2 minutes and 3 minutes in the TAAS group and STEMI group, respectively.

Among the 32 patients in the TAAS group, 20 patients had ST-segments elevation in II, III, AVF leads, and 4 patients combined with level III atrioventricular block. While it made no difference in most types of elevation in the ECG except more patients in the STEMI group could have the V3-V5 leads ST-segments elevation.

### Laboratory test and POCT results

All patients enrolled had point-of-care testing. The median time of reports releasing time was 17 minutes in the TAAS group and 26 minutes STEMI group post PSM analysis. In PSM analysis, patients in the STEMI group had higher positive cTNI (0.174 ng/ml vs. 0.055 ng/ml, P = 0.008) results than the TAAS group, but CK-MB (9.35 ng/ml vs. 5.02 ng/ml, P = 0.151) and Myo (106.00 ng/ml vs. 57.16 ng/ml, P = 0.055) made no significant differences between the 2 groups. In the TAAS group, all the patients had elevated D-dimer results (100% vs. 28.1%, P = 0.000). The results of D-dimer (31.500μg/ml vs. 0.365μg/ml, P < 0.001) and LDH (527.5 U/L vs.350.0 U/L, P < 0.05) in TAAS group were higher than the STEMI group. The concentrations of leukocyte count (WBC), platelet (PLT), hemoglobin (Hb), hs-CRP (CRP), and serum creatinine were not significantly different between the two groups (Table 3).

**Table 3:** TIMEs and lab tests in ED

variable	Pre- PSM			Post-PSM		
	TAAS group (*n =* 32)	STEMI group (*n =* 527)	P-value	TAAS group (*n =* 32)	STEMI group (*n =* 32)	*P*-value
Time description (minutes)						
Time from symptoms onset to admission	85 [55,175]	180 [120,250]	0.000	85 [55,175]	180 [120,300]	0.001
Time from admission to ECG	2 [1,3]	3 [2,5]	0.000	2 [1,3]	3 [2,5]	0.001
Time from admission to D-dimer result	17 [15,26.5]	26 [22,27]	0.000	17 [15,26.5]	26 [21,27]	0.019
Lab test results						
Myoglobin (ng/mL)	296.45 ± 592.13	439.57 ± 560.53	0.000	57.16 [25.5306]	106.5 [56.08,481.25]	0.055
CK-MB (ng/mL)	5.02 [3.11,7.49]	10.61 [3.31,63.93]	0.012	5.02 [3.11,7.49]	9.35 [3.15,54.65]	0.151
LDH (U/L)	527.5 [415.5727]	566 [393,984]	0.602	527.5 [415.52,727]	350 [240,524.50]	0.007
D-dimer (ug/mL)	31.5 [4.77,50.00]	0.44 [0.26,0.83]	0.000	31.5 [4.77,50.55]	0.365 [0.23,0.715]	0.000
Percentage of positive results for D-Dimer (%)	32 (100%)	112 (21.2%)	0.000	32 (100%)	9 (28.1%)	0.000
cTNI (ng/mL)	0.055 [0.008,0.109]	0.177 [0.455,0.87]	0.000	0.055 [0.008,0.109]	0.174 [0.026,0.875]	0.008
WBC (×10^9^/L)	12.00 [9.06,14.00]	10.48 [8.09,13.46]	0.188	12.00 [9.06,14.00]	11.6 [8.53,14.00]	0.941
PLT (×10^9^/L)	256 [219.5293.0]	225 [180,278]	0.018	256 [219.5293]	251 [211.5292]	0.726
Hemoglobin(g/L)	142.5 [129.0,158.5]	140 [129,158]	0.668	142.5 [129,158]	140 [128.5158.5]	0.814
Hs-CRP (mg/L)	3.39 [1.56,15.73]	5.55 [1.92,12.00]	0.682	3.39 [1.56,15.73]	3.71 [1.81,20.63]	0.682
Serum creatinine (umol/L)	89 [67.00,106.00]	71 [56.8,88]	0.015	89 [67,106]	72.9 [56.45,106.8]	0.394

Data are presented as the median [interquartile range], mean ± standard deviation or no. (%) of patients

**Fig. 1:**
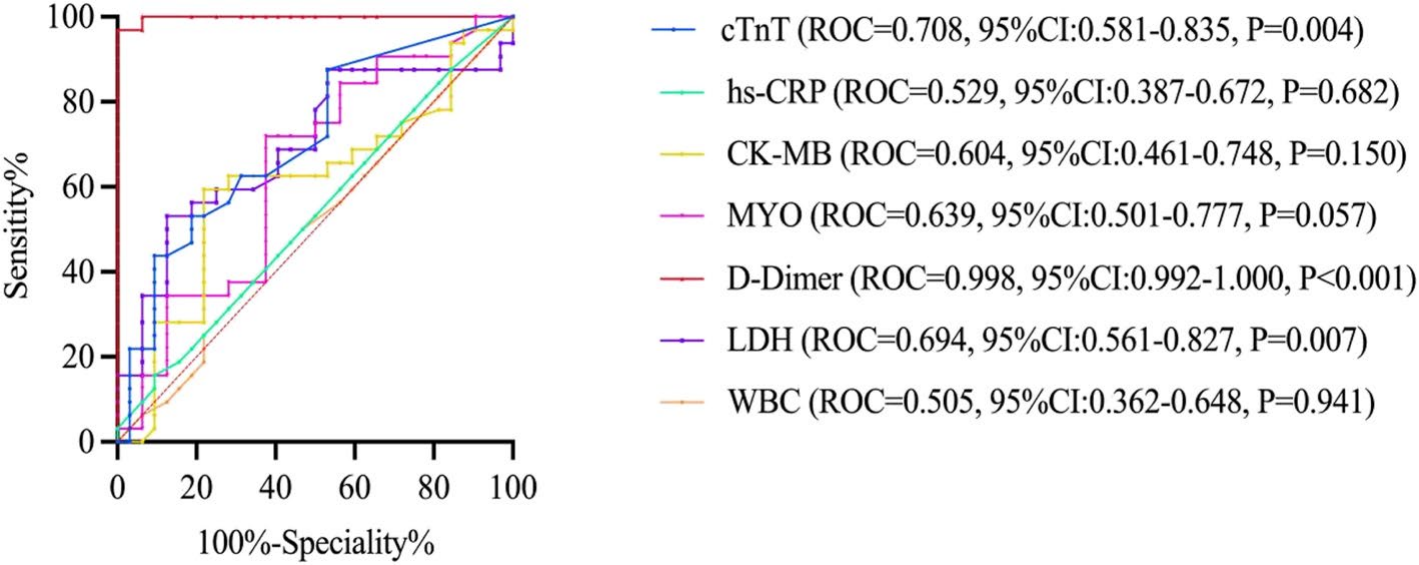
Receiver-operating characteristic curves indicates the cutoff value for D-dimer is set at 2.155μg/ml had the best sensitivity of 100% and specificity of 96.9%

The sensitivities for the total patients of each cutoff value were calculated and used to construct the ROC curves (Fig. 1). The AUC values were 0.998 (95% CI:0.992-1.000, P < 0.001) for the D-dimer, 0.694 (95% CI: 0.561-0.827, *P* = 0.007) for the LDH, 0.708 (95% CI: 0.581-0.835, *P* = 0.004) for the cTNT, 0.604 (95% CI: 0.461-0.748, *P* = 0.150) for the CK-MB, 0.639 (95% CI:0.501-0.777, *P* = 0.057) for the Myo, 0.529 (95% CI:0.387-0.672, *P* = 0.682) for the hs-CRP, 0.505 (95% CI:0.362-0.648, *P* = 0.941) for the WBC.

The D-dimer cutoff value of 2.155μg/ml had the best sensitivity of 100 and 96.9% of specificity, the NPV and PPT of it were 100 and 96.9%, respectively. D-dimer level in the Table 4. showed the cutoff value below 2.15μg/ml exhibited a high NPV, while D-dimer level 2.15μg/ml exhibited a high PPV.

**Table 4:** Different D-dimer cut-off value of diagnostic accuracy (*n =* 64)

D-dimer Cut-off value (ug/ml)	0.6	1.0	1.5	2.15	3.3	4.25	> 4.25
Sensitivity	100%	100%	100%	100%	93.8%	75.1%	68.8%
Specificity	71.8%	87.5%	93.7%	96.9%	93.7%	100%	100%
Positive Predictive value	76.1%	88.8%	91.4%	96.9%	100%	100%	100%
Negative Predictive value	100%	100%	100%	100%	94.1%	84.2%	82.0%
ROC Area under curve	0.968	0.968	0.968	0.998	0.937	0.812	0.75

**Fig. 2:**
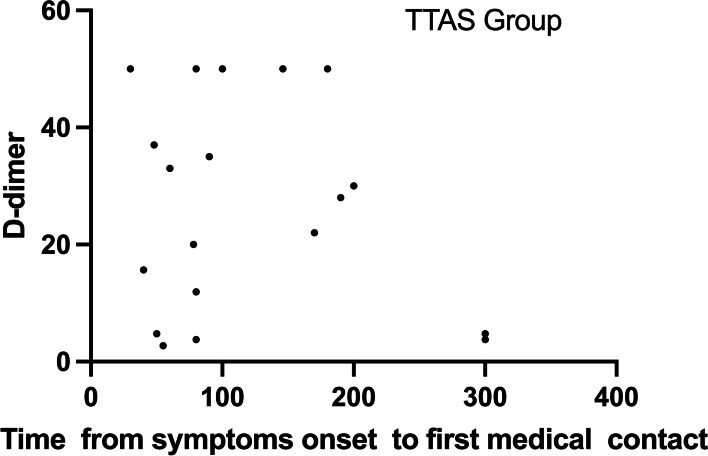
Relationship between D-dimer concentration and TIME (time from the onset of symptoms to first medical contact) in the TAAS group

**Fig. 3:**
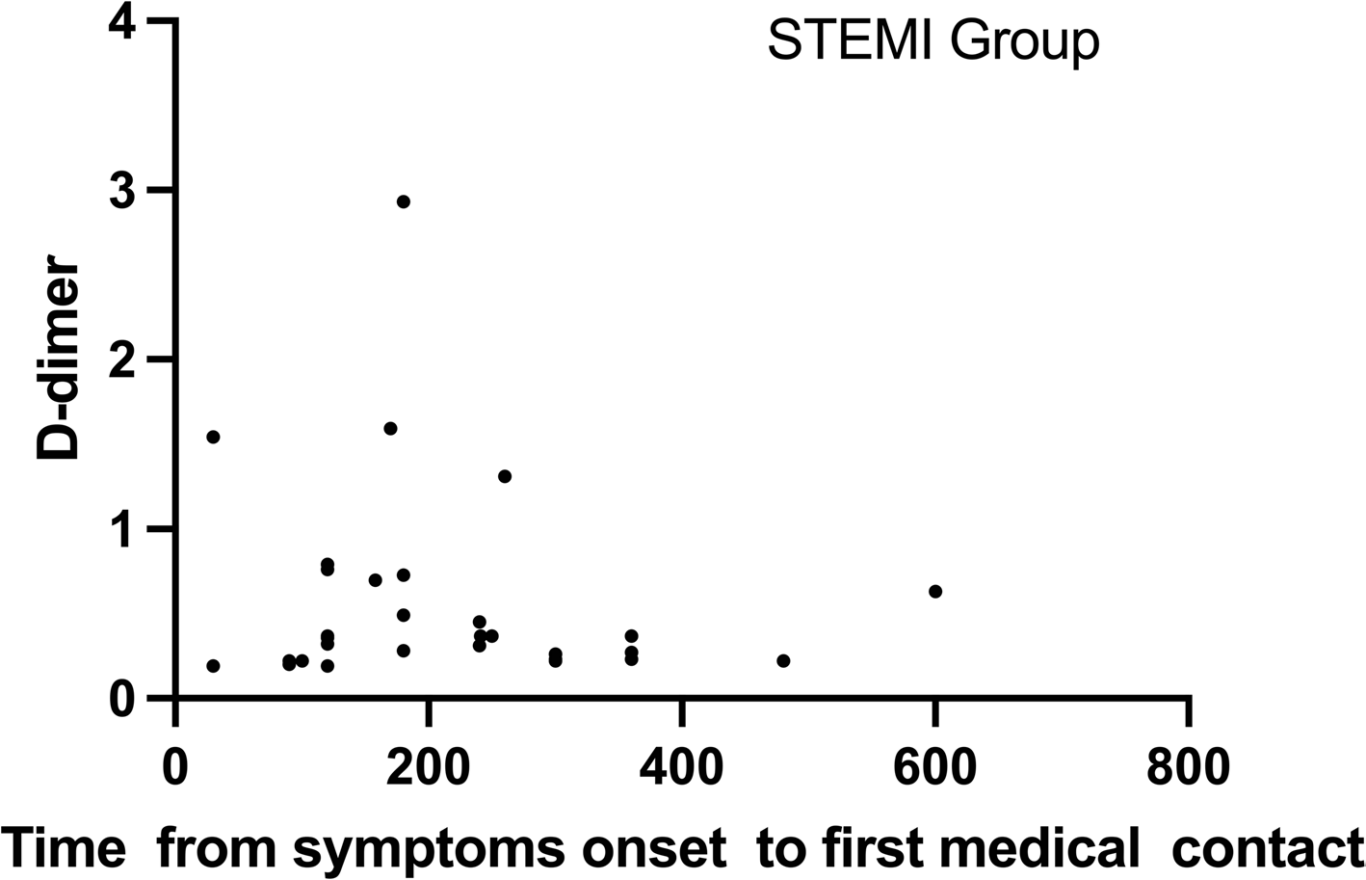
Relationship between D-dimer concentration and TIME (time from the onset of symptoms to first medical contact) in the STEMI group

No significant correlation between the D-dimer concentration and the time from symptom onset to first medical contact in both groups (TAAS group: r = − 0.248, *P* = 0.170; STEMI group: r = − 0.159, *P* = 0.383, Fig. 2. and Fig. 3.). No significant correlation was found between D-dimer and creatinine clearance (TAAS group: r = − 0.065, *P* = 0.765; STEMI group: r = 0.222, *P* = 0.221) as well.

The median time of FMC to CTA confirmation was 41 minutes, while the average time of FMC to CAG was 65.94 ± 3.46 minutes, which is significantly longer than the FMC to CTA confirmation time (*p* < 0.001).

The TAAS group patients were confirmed by the CTA or the digital subtraction angiography (DSA), and the STEMI were confirmed by the DSA. The most common “culprit” artery in regular STEMI patients is right coronary artery (62.5%) followed by the left anterior descending artery which accounts for 31.2%, while in the TAAS group the left main artery is more prone to be affected by the false lumen and the “culprit” artery ECG shows do not coincide with the exact artery affected by the false lumen (Table S1, Fig. S1, Fig. S2 [Supplemental material]).

### Clinical outcomes and Mortality rate of the TAAS patients

In the end, 15 patients underwent CTA and confirmed the diagnosis of TAAS. Among them, 11 patients make it to the surgery and 4 died before the operation. 2 died peri-operation for the cardiogenic shock, and 9 patients survived and discharged, and the 30-day in-hospital mortality was 40%.

**Fig. 4:**
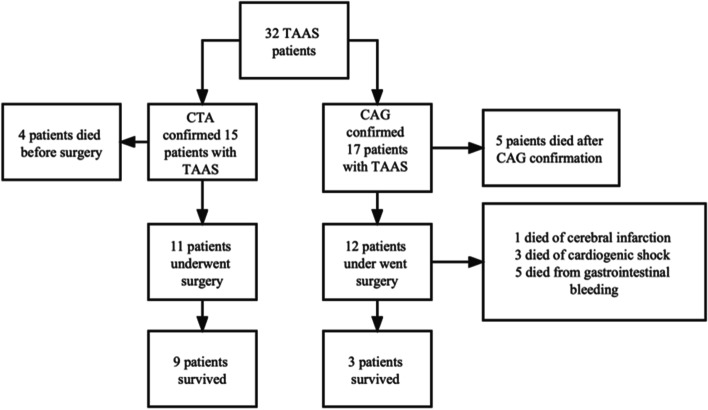
The diagnostic process and outcomes of the TAAS patients

17 patients were given antiplatelet therapy and underwent urgent CAG without awareness of the D-dimer results. 5 patients died after CAG confirmation TAAS without making to emergency surgery for deteriorating cardiogenic shock and malignant ventricular arrhythmia. 12 patients underwent emergency surgery, but only 3 patients survived and were discharged. Among the 9 patients, one patient died on the 17th-day post-operation due to massive cerebral infarction, 3 patients died perioperatively because of cardiogenic shock and multiple organ failure. 5 patients died from uncontrollable gastrointestinal bleeding within 2-4 weeks post-surgery. The clinical data and the outcomes of all 32 TAAS patients were summarized in Table 5. and Fig. 4 .

**Table 5:** TAAS diagnostic methods and time consumption

Variable	CTA (*n =* 15)	CAG (*n =* 17)	*P*-value
TIME (minute)	41 [37,47]	65.94 ± 3.46	0.000
Mortality before surgery	4 (26.7%)	5 (29.4%)	0.897
Cardiac Surgery	11 (73.3%)	12 (70.6%)	1
Peri-operation mortality	2 (18.1%)	9 (75%)	0.006
Cause of death			
Caodiogenic shock	2 (100%)	3 (33.3%**)**	0.695
Cerebral infarction	0 (0%)	1 (11.1%)	0.155
Gastrointestinal hemorrhage	0 (0%)	5 (55.6%)	0.000
30-day in hospital mortality	6 (40%)	14 (82.4%)	0.014
30-day total in hospital mortality	20 (62.5%)		

Data are presented as the median [interquartile range], mean ± standard deviation or no. (%) of patients

TIME, the time from the admission to the ER to the TAAS confirmation

**Fig. 5:**
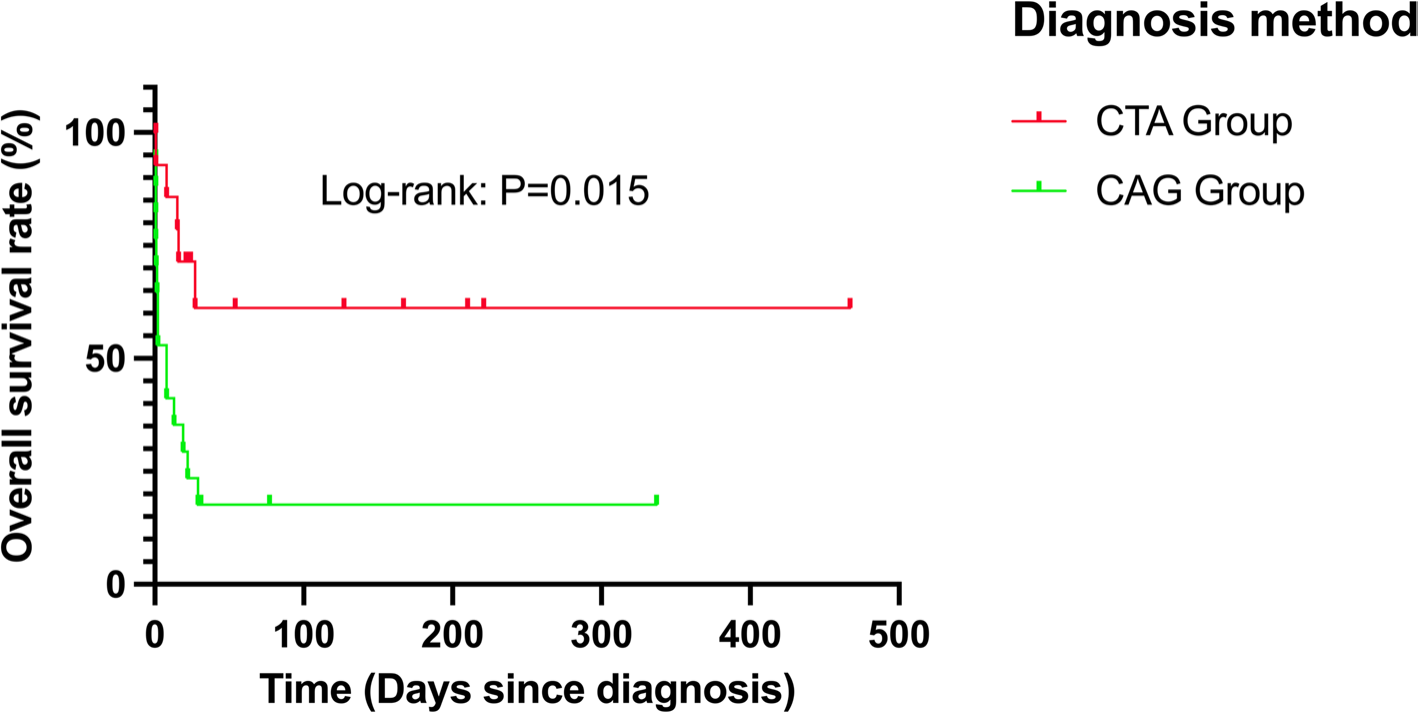
Long-term survival estimates with the use of Kaplan–Meier method after operation for acute type A aortic dissection by diagnostic method. Significant overall difference is observed (*P* < 0.005 by log-rank test)

The total 30-day in-hospital mortality for the patients with TAAS presenting as STEMI was 62.5% (20/32). The 30-day in-hospital mortality rate for TAAS mimicking STEMI receiving CAG was 82.4% (14/17), the 30-day in-hospital mortality rate for TAAS mimicking STEMI receiving CTA was 40% (6/15) which was significantly lower (*P* = 0.014). Long-term survival estimates with the use of Kaplan–Meier method after operation for acute type A aortic dissection by diagnostic method. Significant overall difference is observed (*P* < 0.005 by log-rank test) (Fig. 5).

In the CTA diagnosis group, the average survival time was longer than the CAG diagnosis group with fewer patients who had peri-operation complications, especially gastrointestinal bleeding. CTA diagnosis can reduce the 30-day mortality rate by 67.5% (95% CI:0.124-0.850, *P* = 0.16) (Table 5).

## Discussion

TAAS is rare but a nightmare disease and associated with early mortality rate [6]. Early diagnosis and recognition of the condition is crucial to improve the survival rate. Although the typical patient affected by TAAS can be identified by aortic dissection detection risk score plus D-dimer [7], the patients with atypical features may be misdiagnosed as an uncomplicated regular STEMI [8]. The incidence of acute coronary syndrome secondary to TAAS ranges from 5.7 to 11.3% [9–11] due to acute aortic syndrome of the aortic root reaching the coronary ostia. Approximately 2.5% of patients with TAAS present with STEMI [11] on ECG and misdiagnosis at the emergency department has been shown to be 30 to 78% [7, 8] during the initial assessment. In our study, despite the abnormal result of D-dimer, 53.1% (17/32) patients were misdiagnosed as regular STEMI and more cases of TAAS might be discovered if an autopsy was performed to establish the causes for the mortality among the patients with the diagnosis of STEMI or sudden death.

Clearly, any highly suspicious result in physical examination, laboratory test or supplementary examination with a high level of suspicion and awareness is the key to the timely diagnosis for this special group of patients [9] [12] before being misdiagnosed as regular STEMI and undergoing primary percutaneous coronary intervention. Clinical data including age, sex and clinical manifestations were not helpful in predicting TAAS since the patients with TAAS in our study included both male and female with a wide age range, and chest pain and syncope are the most common presenting initial symptoms [6] in both group. Many studies, including our study, also suggest that some patients who had TAAS mimicking regular STEMI share similar risk factors, including hypertension, aging, sex, diabetes mellitus with uncomplicated STEMI patients [1] [11] [13]. Although there are 49–63% of aortic dissection patients have systolic blood pressure ≥ 150 mmHg upon admission and blood hypertension is also being considered a clinical characteristic of acute aortic syndrome, in our study, the two groups had normal blood pressure (105 mmHg vs. 105 mmHg, *P* = 0.543), thus may lead to overlooking of the rare occurrence of STEMI combined with TAAS when physicians put emphasis on hypertension in TAAS patients. As most physicians agree that ECG with elevated ST-segment and chest pain suffice to diagnose regular STEMI [14] [15], it was certainly possible that some TAAS might have been ignored because of its extremely low odds since over 99% of STEMI is atherosclerotic in nature [16].

CTA scanning is of great value to establish the diagnosis [12]. However, if clinical history and clinical examination may not provide “warning” symptoms, it is very difficult to make the correct and timely differentiate diagnosis, especially in patients with time-sensitive symptoms like cardiogenic shock and malignant ventricular arrhythmia. Emergency CTA is of less value since in primary PCI-capable hospitals, obtaining a CTA imaging could delay the reperfusion therapy.

There was some evidence that aortic dissection may lead to end-organ mal-perfusion syndromes [17] and caused insidious ischemic end-organ complications, which occurred in approximately in one-third of patients [18]. Studies also found that the patients who TAAS underwent CAG who had load dual antiplatelet therapy would have more risk of gastrointestinal hemorrhage [6]. Our research results were consistent with abovementioned results, that TAAS patients confirmed by CAG had more complications peri-operation like gastrointestinal hemorrhage and leading to a higher mortality rate (82.4%) than the published data (23.4–47.7%) from previous studies [1, 19]. As the CAG confirmation time was significantly longer than the CTA confirmation time which may also delay TAAS surgery and increase the risk of mortality.

We found that the rapid bedside D-dimer test gives results in 15 minutes and is useful to screen for TAAS in patients with chest pain combined with ECG ST-segment elevation, and additionally has a D-dimer result of 2.15 μg/ml had the best sensitivity of 100 and 96.9% of specificity.

Serum D-dimer is currently the only clinically proven biological marker available for rapid detection. Contaminated smooth muscle myosin heavy chain [20] and elastin [21] can also be used as biological markers of TAAS with higher specificity, but rapid measurement systems are not clinically available. In general, the fact that D-dimer can be significantly elevated in patients with TAAS is determined by its physiopathological mechanism [22]. And in our study, all patients with TAAS had significantly higher D-dimer elevated over 0.6μg/ml. Additionally, we suspected that the coagulation and fibrinolytic systems are activated immediately after the onset of TAAS, cause no significant correlation between the D-dimer concentration and the time from symptom onset to first medical contact in STEMI group and TAAS group. D-dimer levels have also been reported to correlate with serum creatinine levels [23]. In the present study, there was no correlation between D-dimer levels creatinine clearance in the two groups of patients. Therefore, the cause of elevated D-dimer in patients with TAAS is not impaired renal function, which is an effect of TAAS. Therefore, in the acute phase, a rapid bedside D-dimer test can be used to differentiate between regular STEMI and TAAS combined with STEMI.

Since this special group of STEMI patients has a low incidence and no comprehensive large clinical studies, the management for these patients remains challenging and even controversial. The current guidelines have suggested surgical resection and replacement of the thoracic aorta as the gold standard to treat TAAS [10] [13] [9] [24]. On the other hand, prompt coronary revascularization may do good to the unstable patients, but it can also serve as a double-edge sword and lead to severe complications like refractory hemorrhage peri-operation as in our study. Of course, the management of STEMI caused by TAAS should be individualized. There is no doubt that definitive diagnosis is the most important thing, and up to date, there is no clear guideline to guide the best protocol for distinguishing the diagnosis only the suggestions [25].

Although D-dimer is of high sensitivity and low specificity, and D-dimer concentration tends to be elevated in many other diseases, such as pulmonary embolism [26], deep venous thrombosis [27], cancer [28], atrial fibrillation [29], and congestive heart failure [30]. None of the above diseases are relative contraindications of anti-platelet and reperfusion therapy in STEMI patients, in addition to aortic dissection. If rapid detection shows that the level of D-dimer increases, enhanced CT, transesophageal echocardiography and POCUS are needed to confirm the diagnosis of TAAS before being given antithrombotic therapy. Though the probability of STEMI secondary to TAAS occurrence is very low, patients with more pronounced coronary symptoms and hemodynamic instability may benefit from the fast and accurate diagnosis.

But the authors point out the extreme infrequency, time-sensitive feature, and high mortality rate of STEMI secondary to TAAS in comparison with the total emergency department visits. The prospective systematic approach is difficult to be tested in a clinical situation. Even in the absence of a firm evidence base, the retrospective-based recommendations that the POCT of D-dimer result is important for differential diagnosis between STEMI and TAAS presenting as STEMI are worth consideration. A major strength of the study was to meet the clinical needs in the management of acute TAAS in the emergency department, to minimize time delay in diagnosis and referral of TAAS and increase awareness of the condition and raise the index of suspicion when managing STEMI. The step may only take a few minutes longer, but it may lead to a slightly earlier precise diagnosis and save more lives. To improve on this rather dismal rate, it is worth a try.

### Limitations

The current study has several limitations. This was a single-center experience for differentiating diagnosis of the STEMI and STEMI secondary to TAAS, and as its retrospective nature, it had its disadvantages. The generalization of D-dimer cut-off value is limited due to the small size of the sample, further studies are needed to validate new cut-off values and both the sensitivity and specificity of this approach to a real-world emergency department situation are needed to be tested. In addition to its relatively poor speciality patients with higher D-dimer level still required imaging techniques and may lead to negative TAAS diagnosis and delay reperfusion time. Concerning its good sensitivity, the POCT of D-dimer may thus be a useful rule-out tool. To date, many novel biomarkers have been studied, though no biomarker can reliably identify TAAS as they all have some limitations in terms of sensitivity, specificity or consuming more time, the rapid development of biomarkers over the past decade, may soon allow their incorporation into diagnostic algorithms either alone or in combination with imaging techniques, thus providing clinicians with an additional effective tool as a treatment for STEMI patients secondary to TAAS.

## Supplementary Information

**Additional file 1.**

## Data Availability

The data used to support the finding will not be shared publicly. Researchers can contact the corresponding author for detailed information.
