# Supplementary material for: The point-of-care D-dimer test provides a fast and accurate differential diagnosis of Stanford Type A aortic syndrome and ST-elevated myocardial infarction in emergencies
Source: BMC Cardiovasc Disord. 2022 Dec 21;22:556. doi: 10.1186/s12872-022-02925-x (PMC9768940; doi:10.1186/s12872-022-02925-x)
Supplement: Supplementary file 1 — Additional file 1. [file 12872_2022_2925_MOESM1_ESM.docx]

| Table S1. The “culprit” artery confirmed by DSA or CTA | | | | | | |
| --- | --- | --- | --- | --- | --- | --- |
|  | Pre- PSM | | | Post-PSM | | |
| variable | TAAS group  (n=32) | STEMI group (n=527) | P-value | TAAS group (n=32) | STEMI group  (n=32) | P-value |
| LAD | 3(9.3%) | 221(41.9%) | 0.000 | 3(9.3%) | 10(31.2%) | 0.03 |
| RCA | 19(59.3%) | 232(44.0%) | 0.101 | 19(59.3%) | 20(62.5%) | 0.798 |
| LCX | 0(0.0%) | 61(11.5%) | 0.003 | 0(0.0%) | 1(3.1%) | 1.000 |
| LM | 10(31.2%) | 13(2.4%) | 0.000 | 10(31.2%) | 1(3.1%) | 0.003 |

The “culprit” artery is defined as: a coronary artery affected by a false lumen identified by the surgeon on DSA images in TAAS patients or by the imaging physician on the basis of CTA results; a myocardial infarction-related vessel in STEMI patients.

Data are presented as no. (%) of patients. LM: Left Main Coronary Artery; LAD: Left Anterior Descending; LCX: Left Circumflex ; RCA: Right Coronary Artery.


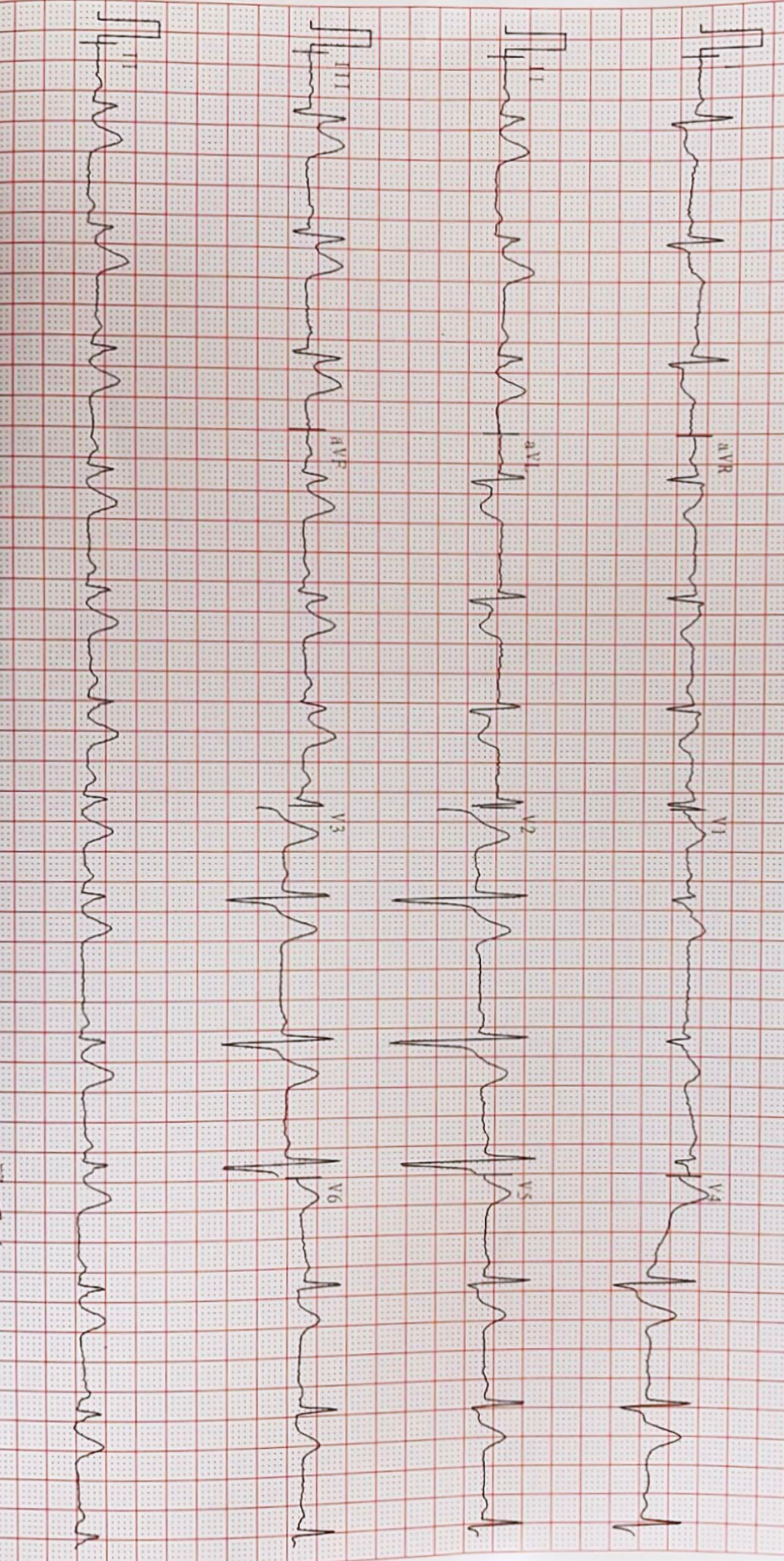


Fig S1: A 65-year-old woman was admitted to the emergency room with epigastric pain as well as vomiting as the chief complaint. The admission electrocardiogram showed II, III, AVF ST-segments elevation.


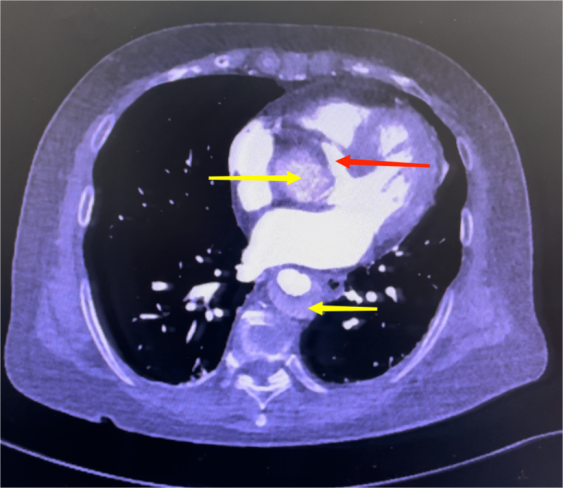


Fig S2: The POCT D-dimer was 4.77ug/ml, and CTA scan in axial scanning whereby aortic dissection involving the RCA ostium (Red arrow) and intramural aortic thrombosis (Yellow arrows) are seen. RCA: right coronary artery
